# Creating Active Device Materials for Nanoelectronics Using Block Copolymer Lithography

**DOI:** 10.3390/nano7100304

**Published:** 2017-09-30

**Authors:** Cian Cummins, Alan P. Bell, Michael A. Morris

**Affiliations:** 1 AMBER Centre and CRANN, Trinity College Dublin, Dublin 2, Ireland; 2 Advanced Microscopy Laboratory (AML), AMBER Centre and CRANN, Trinity College Dublin, Dublin 2, Ireland; bella@tcd.ie

**Keywords:** nanoelectronics, block copolymers, lithography, directed self-assembly, device elements, tungsten trioxide

## Abstract

The prolonged and aggressive nature of scaling to augment the performance of silicon integrated circuits (ICs) and the technical challenges and costs associated with this has led to the study of alternative materials that can use processing schemes analogous to semiconductor manufacturing. We examine the status of recent efforts to develop active device elements using nontraditional lithography in this article, with a specific focus on block copolymer (BCP) feature patterning. An elegant route is demonstrated using directed self-assembly (DSA) of BCPs for the fabrication of aligned tungsten trioxide (WO_3_) nanowires towards nanoelectronic device application. The strategy described avoids conventional lithography practices such as optical patterning as well as repeated etching and deposition protocols and opens up a new approach for device development. Nanoimprint lithography (NIL) silsesquioxane (SSQ)-based trenches were utilized in order to align a cylinder forming poly(styrene)-*block*-poly(4-vinylpyridine) (PS-*b*-P4VP) BCP soft template. We outline WO_3_ nanowire fabrication using a spin-on process and the symmetric current-voltage characteristics of the resulting Ti/Au (5 nm/45 nm) contacted WO_3_ nanowires. The results highlight the simplicity of a solution-based approach that allows creating active device elements and controlling the chemistry of specific self-assembling building blocks. The process enables one to dictate nanoscale chemistry with an unprecedented level of sophistication, forging the way for next-generation nanoelectronic devices. We lastly outline views and future research studies towards improving the current platform to achieve the desired device performance.

## 1. Introduction

Portable electronic devices with enhanced connectivity are ubiquitous in modern everyday life. The use and proliferation of such advanced technology places substantial demands on the often demanding and expensive patterning techniques currently used to satisfy future device and data needs. Conventional top-down lithography practices for patterning logic devices using immersion ultraviolet lithography are meeting expected node milestones through the use of costly multiple patterning processes. Extreme ultraviolet (EUV) technology is anticipated to create sub 7-nm node features; however, such complex routes are proving cost-prohibitive [1]. Thus, cheap, versatile, and compatible strategies such as bottom-up routes are of significant focus for next-generation nanoelectronic devices. Advancing devices with improved performance and functionality has focused scientists on the design, use, and application of nanomaterials for their potential to address society’s ever growing demands. In particular, metal oxide nanofeatures have garnered much interest for nanoelectronic devices due to their enhanced mechanical, chemical, electrical, magnetic, and thermal properties in comparison to their bulk counterparts [2,3]. A major integration hurdle for metal oxide/inorganic fabrication strategies (e.g., vapor-liquid-solid nanowire growth methods) to overcome is their general incompatibility with current silicon technology practices. In this regard, methods that can achieve a trade-off between a material’s superior performance and possess minor risk for industry integration are advantageous. Another issue pertinent to nanoelectronic device performance is the ability for one to dictate the shape, size, and arrangement of metal oxide materials on a wafer. Templating platforms are ideal for one to manipulate the positioning and placement of metal oxide nanomaterials, as many methods exist to enable the replication of an organic template [4,5]. Arranging metal oxides using a soft template may allow the development of superior functional device elements, e.g., resistor, diode, capacitor, etc., in a reasonably economic manner. Block copolymer (BCP) self-assembly offers an excellent route for such templating requirements since sub 50-nm periods and features can be developed over macroscopic areas [6]. Moreover, BCP patterning possesses processing schemes parallel to currently used semiconductor methods [7]. A BCP’s morphology, period size, and ability to microphase separate is tunable through tailoring volume fraction, degree of polymerization, and the Flory Huggins interaction parameter (χ) [8,9]. Critically, directed self-assembly (DSA) of BCPs allows one to assemble features with defined directional alignment when employing physical (graphoepitaxy) [10] or chemical (chemoepitaxy) [11] guiding constraints, an essential facet for device integration.

DSA nanolithography efforts over the past decade have focused on the design/synthesis [12], self-assembly methods [13], alignment [14], and pattern transfer [15] of BCPs for semiconductor translation. On the contrary, little empirical work exists on nanoelectronic device elements developed from mimicking a BCP template or scaffold with an inorganic material. We outline studies that have shown promise hitherto, which gave motivation as well as a new scope for our work. Some of these studies illustrated that complex etch chemistries can be circumvented for developing active device elements that may potentially be useful for interconnect or channel material fabrication. For example, Lopes et al. demonstrated thermally evaporated Ag nanofeatures diffused in PMMA microdomains of a poly(styrene)-*block*-poly(methyl methacrylate) (PS-*b*-PMMA) BCP that exhibited both a non-linear *I*-*V* curve for Ag nanochains and a linear *I*-*V* curve for Ag nanowires, showing their continuity [16]. A solution-based approach was reported by Chai et al., where solutions of anionic metal complexes were used to load metals into PS-*b*-poly(2-vinylpyridine) (PS-*b*-P2VP) BCPs creating Au, Pt, and Pd nanowires [17]. The resistance of individual nanowires was assessed using conductive atomic force microscopy (C-AFM) revealing clear conductivity that was similar to more traditional methods of fabrication. A study by Ross and co-workers [18] also demonstrated the use of C-AFM to assess the electrical conductivity of Pt nanowires that were fabricated through sputtering on an etched PS-*b*-poly(dimethylsiloxane) (PS-*b*-PDMS) film. Similarly, the use of BCPs as a route to pattern arrays for non-volatile memory devices has also emerged [19]. For example, Shin et al. demonstrated multicomponent patterns of different metals such as Au/Pt to achieve a multilevel flash memory device [20].

The approach here offers a truly compatible method compared to vapor-liquid-solid grown nanowires that require extensive post-processing (for pattern placement) and are not compatible for ultra-large-scale integration schemes. While tungsten oxide(s) possess excellent chromic properties, the material’s charge carrying properties have also attracted interest for semiconductor device applications [21]. Tungsten oxide is a distinct metal oxide where phase changes can lead to its use as a semiconductor, conductor, or superconductor, and thus it is chosen due to its promising potential. Moreover, given its compatibility with the metal salt inclusion process, a chloride-based tungsten precursor is used in this study.

We describe the fabrication of well-ordered and aligned tungsten trioxide nanowires templated from a PS-*b*-P4VP BCP. We show how BCP templating offers an efficacious way to include “foreign” metal ion species that can subsequently form metal oxide nanowires with direct nanodevice integration potential. PS-*b*-P4VP BCP was deposited on nanoimprint lithographic graphoepitaxial trenches to align P4VP cylinders under solvent vapor annealing conditions. The aligned P4VP cylinder features were then used for the deposition of a tungsten salt precursor. Nanoscopic and chemical characterization was carried out using scanning electron microscopy (SEM) and X-ray photoelectron spectroscopy (XPS) analysis. The resulting tungsten trioxide (WO_3_) nanowires were subsequently electrically contacted using Ni or Ti/Au material. This article highlights the simple nature of the infiltration process used to create dense functional active device material, and outlines the significant potential that may lie in the method for integration in future nanoelectronics.

## 2. Results and Discussion

**Figure 1:**
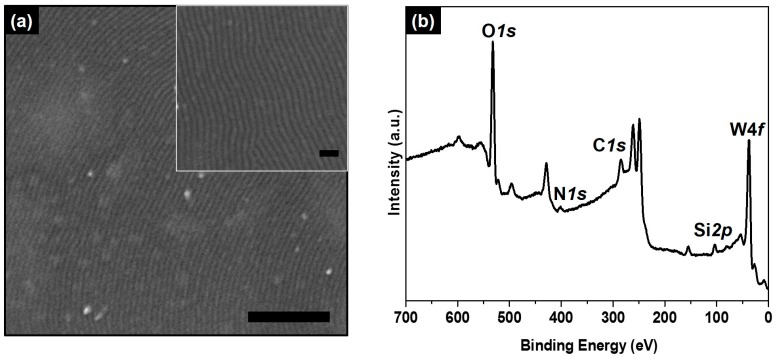

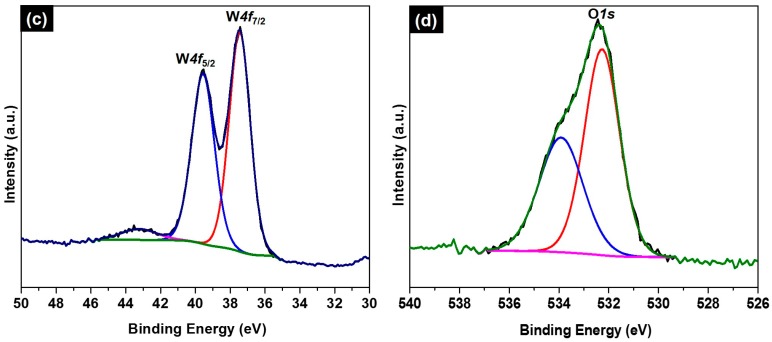
(**a**) SEM image of tungsten trioxide nanowires from PS-b-P4VP line template and tungsten chloride ethanolic precursor post UV/O_3._ Scale bars correspond to 500 nm (main image) and 100 nm (inset), respectively. Corresponding XPS analysis of nanowires showing (**b**) survey spectrum, (**c**) W *4f* high-resolution core scan, and (**d**) O *1s* high-resolution core scan.

To optimize the preparation of tungsten oxide nanowire features for electrical contacting, the sample coverage, nanowire continuity, and chemical composition of the nanowires were assessed prior to translating the process to graphoepitaxial trenches. Solvent vapor annealing in a chloroform atmosphere was employed after the spin-coating of the PS-*b*-P4VP silicon substrates to induce the parallel orientation of P4VP cylinders [22]. The patterns generated were then exposed to ethanol vapors to create nanoporous line space features. This framework was used to create inorganic nanowires from a WCl_4_ precursor, as is discussed in more detail below. Figure 1a shows an SEM image of WO*_x_* nanowires generated from the initial PS-*b*-P4VP BCP scaffold. Large-scale coverage is evident in the SEM images and clear continuity of the nanowires is displayed (see inset, scale bar = 100 nm).

Chemical characterization of the inorganic nanowire material was analyzed using X-ray photoelectron spectroscopy to elucidate the material’s chemical composition. The survey spectrum recorded of the nanowires showed the presence of Si *2p* at ~100 eV (at. % = 9.92%), O *1s* at ~530 eV (at. % = 51.55%), and W *4f* at ~36 eV (at. % = 14.26%), see Figure 1b. The C *1s* region at ~285 eV was detected and the calculated at. % was equal to 21.51%; we attribute this to the contribution from the PS-*b*-P4VP BCP matrix that was not removed during the UV/O_3_ process as well as adventitious carbon. A minor amount of N *1s* at ~400 eV (at. % = 2.75) was also detected, which we attribute to residual P4VP material. The well-resolved high resolution core scan of the W *4f* region is displayed in Figure 1c, showing a doublet peak corresponding to W *4f*_5/2_ at ~39 eV and W *4f*_7/2_ at ~37.5 eV, indicating the formation of W^+6^ in the tungsten oxide material [23]. A notable WO_3_ loss feature is also evident in the core scan for the W *4f* region. The core O *1s* scan (Figure 1d) shows two deconvoluted peaks corresponding to the O atoms in stoichiometric WO_3_ (~532 eV) and the second peak from the O atoms of non-stoichiometric WO_3-*x*_ species (~533.5 eV), in agreement with literature values [24,25]. The O *1s* peak is broader than typically reported and may indicate water molecule content adsorbed on the sample surface.

**Figure 2:**
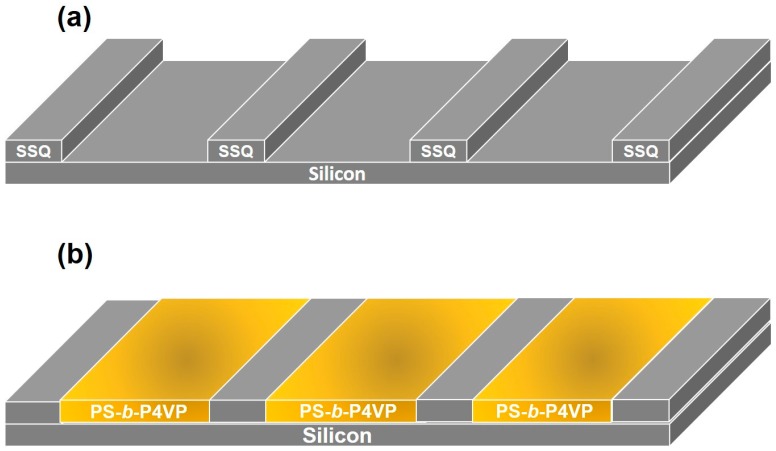

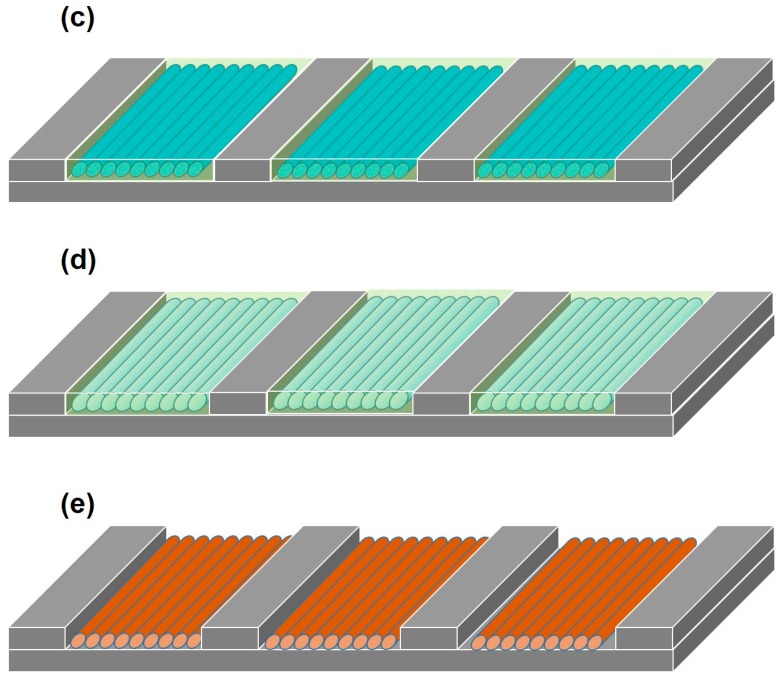
Process flow for developing aligned WO_3_ nanowires from PS-*b*-P4VP BCP. (**a**) Trenched SSQ substrate via nanoimprint lithography. (**b**) Spin-coating of PS-*b*-P4VP block copolymer. (**c**) Line features after solvent vapor annealing. (**d**) Swollen P4VP features after ethanol “activation”. (**e**) Deposition of WCl_4_ precursor and UV/O_3_ process to form WO_3_ nanowires.

The schematic displayed in Figure 2 shows the process for the self-assembly of PS-*b*-P4VP BCP to form line patterns (Figure 2c), followed by ethanol “activation” (Figure 2d), and subsequent WO_3_ nanowire formation (Figure 2e). The process incorporates a nanoimprint lithographically defined trenched substrate, i.e., a graphoepitaxy approach, so that nanofeatures with strict registration and parallel alignment to sidewalls can be formed. Note that previous efforts to electrically contact inorganic nanowires using BCP “fingerprint” patterns proved unsuccessful due to the random nature of the features. Thus, nanowires with continuity over large areas along with alignment are optimum for assessing device characteristics.

**Figure 3:**
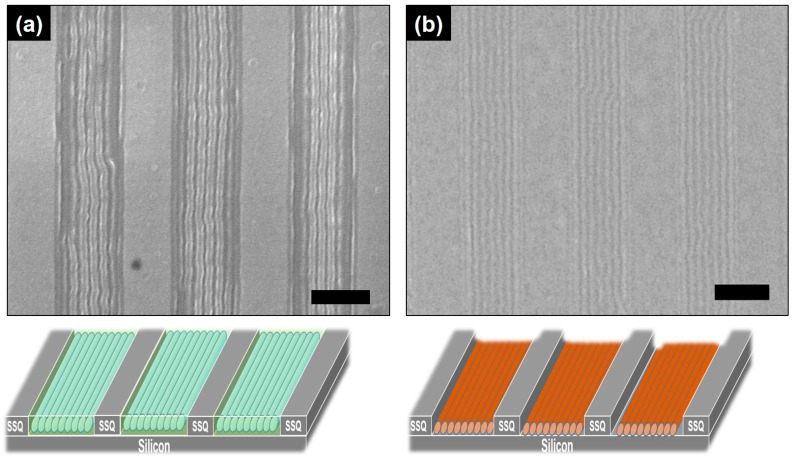
Top-down SEM images of (**a**) aligned PS-*b*-P4VP BCP features after solvent vapor annealing and ethanol “activation” in SSQ trenches nanoimprinted silicon substrates and (**b**) WO_3_ nanowires following metal salt inclusion and UV/O_3_. Scale bars represent 250 nm.

After thin films of PS-*b*-P4VP BCP were deposited on NIL substrates, with a film thickness of ~25 nm as measured through spectroscopic ellipsometry, ethanol exposure (see Materials and Methods section) was carried out in order to develop nanoporous features (Figure 3a). The SEM image in Figure 3a displays the aligned P4VP cylinders parallel to the underlying substrate, which are uniform at silsesquioxane (SSQ) sidewall features through the use of larger trench widths to accommodate excess swelling during solvent vapor annealing [26]. It is critical to examine trench width characteristics for thin films to enable orientation control and alignment [27,28]. Following optimum alignment conditions in our work, the features were used for the incorporation of tungsten metal ions through a spin-on process to develop nanowires mimicking the original PS-*b*-P4VP framework. The swollen P4VP grooves behave as reactive sites for the binding of tungsten ions, and through the loading of a precise concentration, one can tailor the diameter of the resulting nanowires. After the deposition of the WCl_4_ precursor from an ethanolic solution, UV/O_3_ was carried out to eliminate the polymeric template and leave a tungsten trioxide material. We have previously reported the use of this technique to develop hafnia [22], alumina [29], and iron oxide [30,31] amongst other functional inorganics for hardmask fabrication and nanotechnological applications [32]. The resulting WO_3_ nanofeatures are shown in Figure 3b, possessing excellent uniformity and continuity. Nine WO_3_ nanowires with a diameter of ~18 nm and a periodicity of 32 nm are visible within the SSQ trenches.

Previously, Farrell et al. illustrated the promise that DSA PS-*b*-PMMA fabricated silicon nanowires possess for a functional electronic device based on pattern transferred features [33]. The scheme demonstrated here, however, differs significantly since we examine *I*-*V* characteristics of solution-developed WO_3_ nanowires from the PS-*b*-P4VP template at the silicon substrate surface. The platform proposed here is an attractive approach to extending the possibility of utilizing metal oxide nanomaterials emanating from the metal ion infusion of directed BCP patterns without any etching steps required. Moreover, the solution-based method could provide an innovative toolbox for accessing different compositions of metal oxides or inorganics.

**Figure 4:**
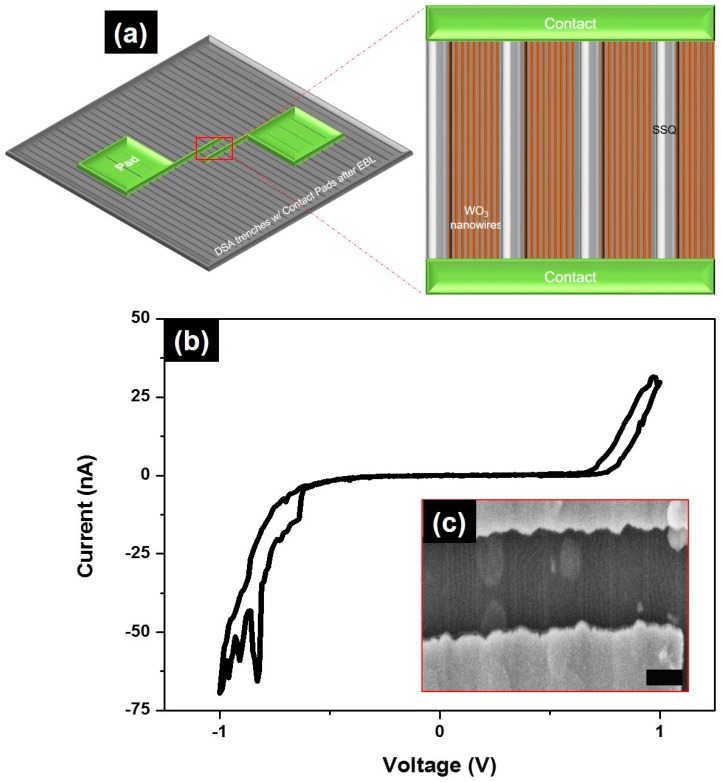
(**a**) Scheme showing large scale area of contact pads on SSQ patterned substrates containing WO_3_ nanowires within trenched features from the initial PS-*b*-P4VP BCP template. (**b**) *I*-*V* curve measured on WO_3_ nanowires contacted with Ni pads. (**c**) Inset shows aligned WO_3_ nanowires within trenches; scale bar represents 200 nm.

We examined the *I*-*V* characteristics using different metals for contacting the aligned WO_3_ nanowires. Initially, contact pads composed of Ni (50 nm) deposited through e-beam were fabricated on e-beam lithography developed patterns across multiple trenches of WO_3_ nanowires (see scheme in Figure 4 for layout). Channel widths for the Ni contact pads on aligned WO_3_ nanowires were ~400 nm and ~5 μm in length, and the SEM inset reveals the impressive order of the WO_3_ nanowires. Initial *I*-*V* results using this approach revealed relatively consistent data of two diodes in series, with a characteristic symmetric *I*-*V* curve shown in Figure 4, for a voltage of ±1 V. The performance of the material observed suggests that the nanowires are indeed continuous, even over large areas, and that few large defects exist in the nanowires. Symmetric *I*-*V* curves as observed here indicate the formation of Schottky barriers between the Ni contacts and the WO_3_ nanowires, and the nanowires are thus semiconducting. The results are extremely similar to a report by Rui et al. on chemical vapor deposited tungsten oxide (W_18_O_49_) nanowires, where *I*-*V* measurements were measured using an in situ transmission electron microscope method [34]. The approach used was extremely complex with respect to the VLS nanowire synthesis involved as well as the contacting method for *I*-*V* analysis. In contrast, despite using alternative lithography practices in our study, a compatible semiconductor processing scheme was implemented to attain features that performed relatively similarly to nanowires grown through vapour-solid mechanisms. While the results proved to be promising as an initial test, the current measured was quite low for Ni contacts, and thus alternative metals were processed in order to better analyze the channel material and form low resistivity contacts.

**Figure 5:**
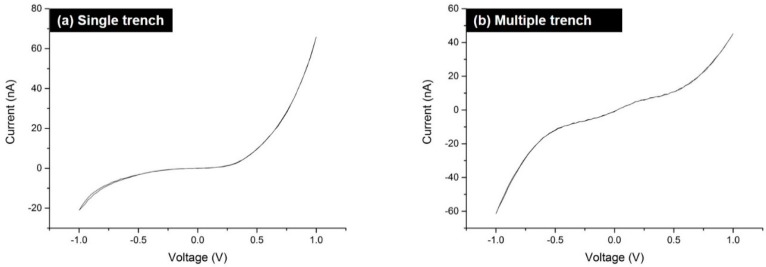
*I*-*V* curves of contacted WO_3_ nanowires in (**a**) single trench and (**b**) multiple trenches. See text for further details.

Subsequently, Ti/Au (5 nm/45 nm) contacts were fabricated in the same manner, using electron beam lithography (EBL), as used for the Ni contacts shown above. Ti/Au are routinely employed as contact material structures [35]. To obtain a better understanding of how the number of channels affected the results, measurements were taken on both single channel areas, i.e., nine WO_3_ nanowires, and on five trenches containing arrays of WO_3_ nanowires. Thirty devices were fabricated, with current recorded in 15 devices. A higher current was recorded for both devices in comparison to the Ni contacts described above, with typical *I*-*V* currents displayed in Figure 5a (single trench, ~60 nA at 1 V) and Figure 5b (5 trenches, ~50 nA at 1 V). Resistivity was calculated as low as 10 MΩ cm for both the trench types analyzed. The current measured across the single trench of WO_3_ nanowires is marginally higher than the multiple trench. We believe the lower current may result in the multiple trench of WO_3_ nanowires for a number of reasons. Firstly, shards of the contacts from the deposition process on the mesas of the NIL imprint may be a contributing factor to the lower current. Moreover, alignment defects of the 1-D nanowires as well as the nanowires continuity over large areas, i.e., five trenches, may have had a greater influence on the lower current measured compared to continuous nanowires from a single trench alone. Given that the electrical test data described here represents a pilot study, further measurements are needed in order to statistically quantify parameters including (i) WO_3_ nanowire alignment/order within contacts and (ii) single trench nanowire versus multi-trench nanowire devices.

The results discussed above present a significantly promising strategy for the development of active device material for nanoelectronic devices centred on scalable alternative lithography methods. It is clear that the DSA methods outlined are compatible to semiconductor manufacture, since the complex engineering of nanowires inherent to other bottom-up self-assembly routes [36] is not required. While the DSA BCP field has burgeoned over the past decade, reports of a real solution or demonstration of nanowires/vias structures that can be utilized in integrated circuit manufacture have been scant. As evidenced from the data presented, this simple processing approach offers many opportunities for the selective inclusion of functional materials in nanoarchitectures for device use. Next, we discuss future research directions that will serve as a basis to assess the prospect of using BCP lithography and metal ion infusion techniques to create a nanoelectronic device architecture in an etchless fashion.

The electrical testing of inorganic nanowires replicated from BCP templates is in its infancy. Thus, we outline some milestones to drive the area towards functional nanoelectronic devices based on our work described above. Firstly, carrying out studies on the electron transport properties of the nanowires more thoroughly would be beneficial. Studies should encompass in-depth analyses of reduced nanowires, e.g., Ni or Cu nanowires. Secondly, examining the electrical performance of such nanowires as a function of varying nanowire diameters is critical. Manipulating the nanowire diameter is relatively facile, and can be achieved by changing the respective BCP molecular weight or fine-tuning the metal salt precursor concentration. Such dynamic evaluation through electrical studies will be key to validating the overall feasibility of the salt inclusion process for device integration. Future research should also focus on the study of the channel material fabricated. This could prove to be extremely fruitful for researchers since the metal salt inclusion process is amenable to a plethora of salt precursors. The salt inclusion strategy can enable “traditional” semiconductors, and more attractive features such as ferroelectrics may be of interest for nanoelectronics. Access to a wide scope of material sets is possible using the metal salt inclusion technique. Aside from channel material analysis, key parameters to expand the knowledge of the work here should center efforts on improving the gate development process, on the evaluation of metal contacts, and on the study of the top gating of metal oxide 1-D nanowire features to make fin field effect transistor (FinFET) type devices.

## 3. Materials and Methods

### 3.1. Materials 

Poly(styrene)-*block*-poly(4-vinylpyridine) (referred to as PS-*b*-P4VP BCP herein) was purchased from Polymer Source, Inc., Dorval, QC, Canada, with a molecular weight of Mn = 33.5 kg mol^−1^ (Mn_PS_ = 24 kg mol^−1^; Mn_P4VP_ = 9.5 kg mol^−1^, fPS = 0.70) and a polydispersity (Mw/Mn) of 1.15 (where Mn and Mw are number average and weight average molecular weights), and was used as received. WCl_4_ (tungsten (IV) chloride), toluene, chloroform (for HPLC, ≥99.9%, contains 0.5–1.0% ethanol as stabilizer), tetrahydrofuran (inhibitor-free, CHROMASOLV Plus, for HPLC, ≥99.9%), toluene (for HPLC, ≥99.9%), and ethanol (dehydrated, 200 proof) were purchased from Sigma-Aldrich (Darmstadt, Germany) and used without further purification unless otherwise stated. Deionized (DI) water was used wherever necessary. Blanket Si (provided by Intel, Santa Clara, CA, USA) and nanoimprint lithography (NIL) silsesquioxane (SSQ) substrates (provided by collaborators) were employed in this study. The graphoepitaxy substrates were prepared on silicon substrates and were fabricated using UV (ultraviolet) nanoimprint lithography (Laboratoire des Technologies de la Microelectronique (CNRS), Grenoble, France) with elastomeric molds. Further details on NIL fabrication can be found in Reference [37].

### 3.2. Film Deposition and Nanowire Formation

PS-*b*-P4VP solutions were prepared at 0.5 wt % concentration in toluene/tetrahydrofuran (80:20) and stirred until clear. Polymer solutions were spin-coated (30 s, 3200 rpm) on NIL patterned substrates creating a film with a thickness of ~25 nm. Films were solvent vapor annealed [38] with a small vial containing 8–10 mL chloroform placed inside a glass jar (150 mL) for 1 h, as reported previously by our group, for SVA on silicon substrates [29]. The samples were then “activated” via exposure to ethanol vapors at 50 °C for 20 min from a nanoporous matrix. A 1% tungsten chloride ethanolic solution was subsequently spin-coated (30 s, 3200 rpm) on the nanoporous matrix followed by UV/O_3_ treatment for 3 h (UV/ozone system—PSD Pro Series Digital UV Ozone System; Novascan Technologies, Inc., Ames, IA, USA).

### 3.3. Electrical Studies

After tungsten oxide nanowire formation, an array of metal contact pads was deposited for electrical measurements. Electron Beam Lithography (EBL) was employed to contact the aligned nanowires. PMMA A3 was spin-coated at 3000 rpm to give a resist thickness of ~150 nm. The film was baked at 180 °C for 3 min. Following this, samples were loaded into a Zeiss SUPRA SEM (Carl Zeiss AG, Oberkochen, Germany) with a Raith Elphy (Raith GmbH, Dortmund, Germany) attachment. Exposures took place using an accelerating voltage of 15 kV and beam current of 400 pA. Doses of 400 μC·cm^−2^ were used to expose the resist. Resist development took place in chilled 1:3 (*v*:*v*) methyl isobutyl ketone: isopropyl alcohol (MIBK:IPA) for 30 s, IPA was used as a stopper. The subsequent metallization step was carried out in an e-beam evaporation system (Temescal FC-2000, Ferrotec, Livermore, CA, USA). Two contact systems were investigated, and this article details the use of Ni only (~50 nm); the second system was a Ti/Au contact (5 nm of Ti and 45 nm of Au). A two-point probe system (Keithley 2400, Keithley Instruments, Cleveland, OH, USA) was used to measure current against the voltage applied.

### 3.4. Characterization

PS-*b*-P4VP BCP film thicknesses were measured with a spectroscopic ellipsometer (J. A. Woollam Ellipsometer, Lincoln, NE, USA) at a fixed angle of incidence of 70°, on at least five different places on the sample, the results of which were averaged as the film thickness. A two-layer model (SiO_2_ + BCP) for total BCP film was used to simulate the experimental data. Top-down scanning electron microscope (SEM) images were obtained by a high resolution (<1 nm) Field Emission Zeiss Ultra Plus-Scanning Electron (Carl Zeiss AG, Oberkochen, Germany) with a Gemini^®^ column operating at an accelerating voltage of 5 kV. Domain size and periodicity of feature sizes were measured from SEM data using ImageJ software (open platform, https://imagej.nih.gov/ij/). X-ray Photoelectron Spectroscopy (England, UK) was performed under ultra-high vacuum conditions (<5 × 10^−10^ mbar) on a VG Scientific ESCAlab Mk II system using Al Kα X-rays (1486.6 eV). The analyzer pass energy was set to 100 eV for survey spectra and 20 eV for high-resolution core scans when recorded. Photoemission peak positions were corrected to a C *1s* feature at a binding energy of 284.8 eV.

## 4. Conclusions

This work demonstrated a facile and semiconductor-compatible strategy towards an active functional nanoelectronic device without conventional lithographic fabrication methods. The methodology described is a robust approach to creating 1-D nanowire channel materials for nanoelectronic application. A synergistic directed self-assembly approach using NIL SSQ trenches and PS-*b*-P4VP BCP features generated parallel aligned line-space features to trench sidewalls. A wet chemical spin-on process was then optimized to generate WO_3_ nanowires, which were subsequently contacted to assess I–V characteristics. The electrical results of the WO_3_ nanowires presented show promise for advancing BCP-metal ion solution-based nanowire fabrication methods into ultra-large-scale integration schemes. Ti/Au contacts proved to be a better contact structure compared to Ni from the measured current. In summary, we have shown a cost-effective alternative to conventional lithography for developing a functional device through DSA and metal ion infusion of BCPs. We envision the extension of this technique to produce other active device materials, e.g., phase change nanowires that can fulfil future nanoelectronic device demands.
